# Direct measurements of IPTG enable analysis of the induction behavior of *E. coli* in high cell density cultures

**DOI:** 10.1186/1475-2859-11-58

**Published:** 2012-05-09

**Authors:** Alfred Fernández-Castané, Glòria Caminal, Josep López-Santín

**Affiliations:** 1 Departament d’Enginyeria Química, Unitat de Biocatàlisi Aplicada associada al IQAC (CSIC), Universitat Autònoma de Barcelona, Bellaterra, Spain

**Keywords:** IPTG transport, Recombinant protein production, Fed-batch, Bistability, Permease

## Abstract

**Background:**

The *E. coli lac* operon and its components have been studied for decades, and *lac*-derived systems are widely used for recombinant protein production. However, *lac* operon dynamics and induction behavior remain the paradigm of gene regulation. Recently, an HPLC-MS-based method to quantify IPTG in the medium and inside the biomass has been established, and this tool may be useful to uncover the lack of knowledge and allow optimization of biotechnological processes.

**Results:**

The results obtained from the study of IPTG distribution profiles in fed-batch, high cell density cultures allowed discrimination between two different depletion patterns of an inducer from the medium to the biomass in *E. coli*-expressing rhamnulose-1-phosphate aldolase (RhuA). Moreover, we could demonstrate that active transport mediates the uptake of this gratuitous inducer. Additionally, we could study the induction behaviors of this expression system by taking into account the biomass concentration at the induction time.

**Conclusions:**

In the bistable range, partial induction occurred, which led to intermediate levels of RhuA activity. There was a direct relationship between the initial inducer concentrations and the initial inducer transport rate together with the specific activity. A majority of the inducer remains in the medium to reach equilibrium with the intracellular level. The intracellular inducer accumulation was a further evidence of bistability of the *lac* operon.

## Background

Although many experimental studies have been carried out for over 50 years and several mathematical models have been proposed for describing the *lac* operon regulation, the *lac* operon remains the paradigm of gene regulation [1-3]. Currently, *lac* operon regulation is a research objective in systems biology, and *lac* operon elements are used as tools in biotechnology and synthetic biology [4-7]. For this reason, a detailed knowledge about the *lac* operon induction behavior is required.

However, no methods have been available to quantify IPTG (Isopropyl-β-D-1-thiolgalactopyranoside) in the medium or inside the biomass, and assumptions have been made using just the initial concentration of the inducer added to the culture and using reporter genes or β-galactosidase activity to give quantitative approaches on *lac* operon dynamics.

Early studies of the *lac* operon, which were based on the pioneering work of Cohn & Horibata [8,9], were concerned with the kinetics of enzyme induction in the presence of gratuitous inducers, such as TMG (thiomethyl galactosides) and IPTG, which cannot be hydrolyzed. These early studies showed that the enzyme synthesis pattern was bistable, with pre-induced cells remaining induced and non-induced cells remaining non-induced [8,9]. Furthermore, this bistability disappeared in cryptic mutants lacking *lac*-permease (lactose permease) [10]. It was proposed that the bistability occurred because of the destabilizing effect of the positive feedback generated by *lac*-permease that may catalyze the accumulation of the inducer, which in turn stimulates the synthesis of even more permease. Conversely, lactose, the natural inducer of the *lac* operon, stimulates not only the synthesis of the *lac* operon enzymes but also cell growth because it is used as a carbon source [11-13]. Recently, Ozbudak et al. [14] performed detailed studies of *lac* bistability, and they observed that cells exhibited bistability when grown in the presence of succinate and various concentrations of TMG. Moreover, the bistability persisted even if glucose was added to medium containing succinate and TMG, but the thresholds increased with the concentration of extracellular glucose. Laurent et al. [15] developed a mathematical model to explain the bistability of the *lac* operon and concluded that IPTG may be transported by *lac*-permease, and therefore, both the medium and intracellular inducer concentrations should be considered. Other recent works stated that IPTG could enter by *lac*-permease but that a *lac*-permease-independent entry was possible [16]*.*

However, to understand the induction kinetics, the molecular basis of the *lac* operon and IPTG induction must be considered [17-19]. In a wild type strain, the *lac* operon contains two auxiliary operators, O2 and O3 (in addition to the main operator), and the repressor is a tetramer containing four inducer-binding sites [19]. Furthermore, repression can also be caused by the formation of DNA loops rather than the repressor-operator binding [20,21]. In addition, the bioreactor mixing efficiency may affect the cell population behavior [22].

Noel et al. [23] conducted simulations of kinetic models using the experimental data from Ozbudak and Laurent to study the inducer transport phenomena. It was proposed that carrier efflux could not be ignored for induced cells and diffusive influx could not be neglected for non-induced cells in experiments measuring β-galactosidase activity [23], and the idea that *lac*-permease catalyzes IPTG transport is still controversial.

Nevertheless, few studies in the literature take into account the effect of the biomass concentration in the culture when inducing the *lac* operon, and none account for high cell density cultures. This fact may be of paramount importance when producing heterologous proteins in high cell density cultures. Moreover, no direct measurement of IPTG has been provided to support the hypotheses of the previous studies.

We have recently established an HPLC-MS-based method to measure medium and intracellular IPTG in fed-batch culture samples under substrate-limiting conditions [24]. Moreover, we have demonstrated that *lac*-permease mediates IPTG transport, and when this transporter is lacking, diffusion is the responsible transport mechanism [25].

In this work, we provide quantitative data of medium and intracellular IPTG concentration evolution in high cell density fed-batch samples in an expression system derived from the *lac* operon. Distribution profiles of IPTG in the medium as well as the intracellular accumulation of the inducer were analyzed and discussed to provide a better understanding of *lac*-derived operon dynamics and transport phenomena that are likely involved in the induction of rhamnulose-1-phosphate aldolase (RhuA) expression at different levels of IPTG. Furthermore, we aimed to correlate the induction behavior with the maximum RhuA activity achieved in each experiment, in which we considered that the medium and intracellular inducer concentrations reached equilibrium (“pseudo-steady” state). Our methodology does not provide direct evidence of the bistability of the *lac* operon but intends to explain the induction patterns of the recombinant protein RhuA in high cell density fed-batch cultures and the possible elements involved.

## Results and discussion

### The effect of the biomass concentration

As stated in the background section, previous studies carried out on the dynamic behavior of the *lac* operon do not provide experimental data for IPTG and do not report the biomass concentration upon induction in high cell density cultures. This fact should be considered a key parameter because inducer uptake may be affected by the number of cells capable of incorporating the inducer within the cytoplasm. These early experiments were performed on a shake flask scale with a low concentration of biomass. In contrast, this work addresses an auxotrophic expression system for production of RhuA in fed-batch cultures using a predefined exponential feeding profile to reach biomass concentrations at production scales. Moreover, one has to take into account that this system harbors two plasmids (pQEαβrham and pREP4) expressing the LacI protein constitutively and has more operator sites than the wild type strain. Plasmid copy number of pQEαβrham and pREP4 is 30–40 and 10–12, respectively [26].

Therefore, it was convenient to assess the influence of cell concentration on IPTG uptake. Table 1 summarizes the results obtained for RhuA production in terms of specific activity and initial transport rate values (q_I0_) for two different biomass concentrations and inducing RhuA with IPTG at 20 and 100 μM. Using the same inducer concentration, lower specific activities were obtained upon induction at 40 g DCW ·L^-1^ than when the biomass concentration at induction was 20 g DCW ·L^-1^. Similar results were obtained for the two inducer levels studied. Table 1 also shows a clear dependency of the initial uptake rate of IPTG from medium on the biomass concentration at induction. The higher biomass leads to a faster entry of IPTG into the cell. Therefore, the biomass concentration of a culture should be considered when studying *lac* operon dynamics in cell populations. Consequently, the degree of induction not only depends on the environmental conditions, i.e., IPTG concentration in the medium but also on the concentration of the biomass population that may compromise the inducer availability for a single cell at low concentrations of the inducer.

**Table 1:** **Comparison of RhuA production and transport rate values (q**_**I0**_**)**

**Ferm.code**	**[IPTG]**_**0**_**(μM)**	**X**_**ind**_**(g DCW·L**^**-1**^**)**	**RhuA activ. (AU·g**^**-1**^** DCW)**	**q**_**I0**_**(μM·h**^**-1**^**)**
FB3	20	20	769	5
FB3B	20	40	380	1478
FB6	100	20	1028	350
FB6B	100	40	513	2141

### Distribution profiles of IPTG in medium and inside the biomass: induction behavior

**Figure 1:**
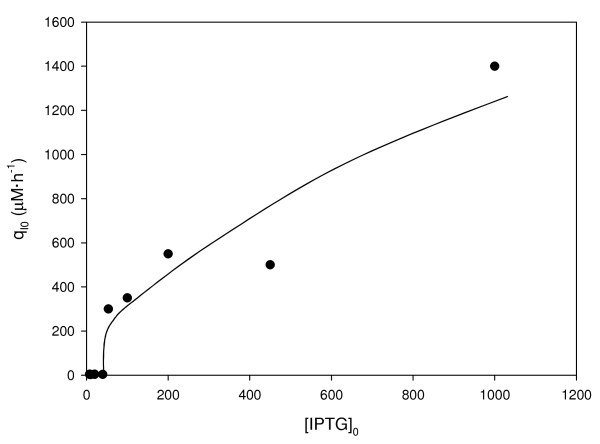
**Initial IPTG transport rate (q**_**I0**_**, μM·h**^**-1**^**) from medium to the biomass vs. initial inducer concentration (IPTG**_**0**_**, μM) in fed-batch substrate limiting experiments.** Each point corresponds to the initial slope of IPTG medium concentration profile.

A study of the induction behavior using fed-batch experiments was performed under the same conditions, with the only variation being the initial inducer concentration (8–1000 μM). We could observe two different patterns of IPTG depletion from the medium upon induction (see Additional file 1). In fermentations induced with up to 40 μM, IPTG decreased from the medium gradually, resulting in initial transport rate (q_I0_) values of 3–5 μM·h^-1^; however, upon induction at higher concentrations, IPTG is initially depleted from the medium rapidly, resulting in initial transport rates of 300 to 1400 μM·h^-1^ .q_I0_ values were calculated as the initial slope of the extracellular IPTG profiles (see Additional file 1), which are shown in Figure 1. The number of sonication cycles for disruption of biomass samples and subsequent release of intracellular inducer was assessed. After four sonication cycles maximum IPTG levels were measured. The increase of the number of sonication cycles within a sample did not lead to a further release of the inducer. Distribution profiles of intracellular IPTG at different initial inducer concentration levels at induction time were evaluated. The intracellular inducer concentration increases up to a certain concentration, although, in certain cases, the intracellular IPTG first increases and then decreases.

The two differential patterns of inducer depletion from the medium can be attributed to the different mechanisms of transport involved in IPTG uptake. The cases in which intracellular IPTG increases to a certain concentration and then decreases could be due to the feedstock supply to the reactor and to the increase of cell volume with cellular division. Additionally, cell lysis could promote the release of the inducer into the extracellular medium. The employed analytical method for IPTG can only measure free IPTG, and not the one bound to other molecules.

**Figure 2:**
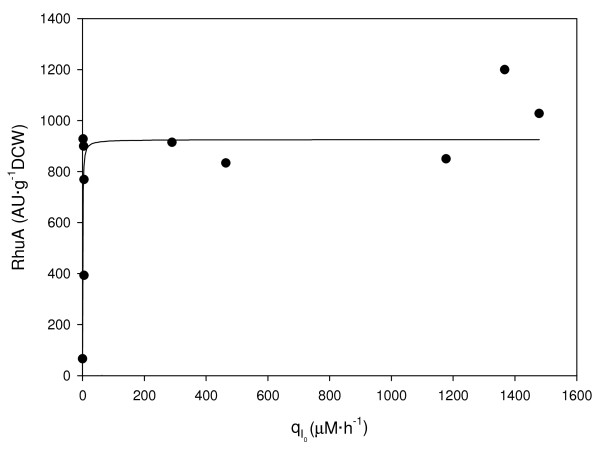
**Dependency of specific RhuA activity (AU·g**^**-1**^**DCW) at “pseudo-steady” state on the initial inducer transport rate (q**_**I0**_**, μM·h**^**-1**^**) from medium to the biomass.**

**Figure 3:**
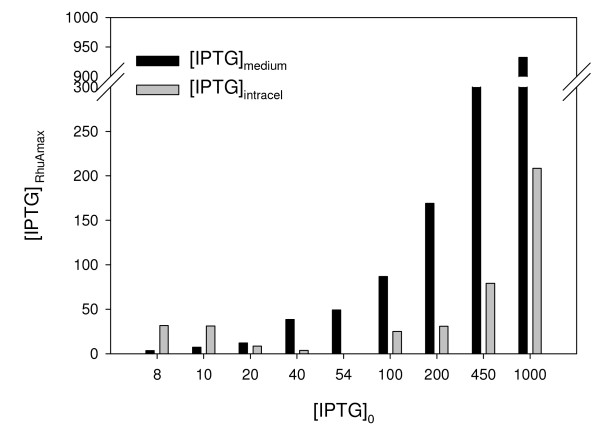
**Comparison of medium and intracellular IPTG concentrations (μM) when specific RhuA (AU·g**^**-1**^**DCW) is maximum (“pseudo-steady” state) at different initial inducer concentrations (IPTG**_**0**_**, μM).** The analytical standard error for experimental IPTG measurements is 5 and 7% for medium and intracellular samples, respectively.

A comparison between the initial transport rate and specific activity values at the pseudo-steady state was performed. The results shown in Figure 1 indicate that low q_I0_ values were obtained at initial inducer concentrations corresponding to 8 to 40 μM IPTG and that coincided with intermediate levels of induction. The maximal activity of recombinant protein was obtained above 40 μM IPTG, which coincided with higher q_I0_ values (Figure 2). Therefore, we suggest that the full induction of RhuA is achieved above 20 to 40 μM IPTG. According to previously published results [25], we postulate that *lac*-permease has a lesser role at low initial inducer concentrations (partial induction) than at relatively high values of initial inducer concentration. Our results are in agreement with the theoretical model of the dynamic behavior of the *lac* operon that was proposed by Laurent [15]. It is likely that above a threshold value, which was 40 μM IPTG in our system, the transcription of permease is completely triggered, consequently enhancing the uptake of the inducer, and subsequently, RhuA activity reaches its maximum values. Nevertheless, active transport may occur at all of the inducer concentrations studied because the measured intracellular concentrations of inducer IPTG_0_ (8–10 μM) were higher than in the medium at the “pseudo-steady” state, as shown in Figure 3.

### Comparison of the medium and intracellular IPTG concentrations in the “pseudo-steady” state

The concentrations of the inducer in the medium and intracellular are shown in Figure 3, when RhuA was at a maximum. As anticipated, upon induction at 8–10 μM IPTG, the intracellular concentration of the inducer was higher than that in the medium, but reached similar values at 20 μM IPTG. At higher initial concentrations of IPTG, the medium values were always higher than those inside the biomass.

When there was no inducer, [IPTG]_intra_ = [IPTG]_extra_ = 0, a steady state (SS1). In our expression system, we can assume that at low concentrations of IPTG, the initial inducer concentration is so low that it cannot enter the cell ([IPTG]_intra_ < [IPTG]_extra_). From previous experiments performed by our research group, we know that there is no RhuA overexpression upon induction at IPTG concentrations lower than 4 μM [26]. By increasing the extracellular inducer, IPTG can enter the cell by diffusion and/or active transport and can accumulate until it reaches a second steady state (SS2) at concentrations ≤ 4 μM, being SS2 the “on” threshold to induce RhuA. Then, the intracellular concentration overcomes the medium concentration at 8–10 μM ([IPTG]_intra_ > [IPTG]_extra_). At this stage, RhuA expression is not fully induced because the intracellular inducer does not bind the repressor with an affinity strong enough to release all of the repressor molecules from all of the operator sites. Another possible explanation is that there are some cells induced and others remain non-induced when using low concentrations of IPTG. By increasing the initial inducer concentration, the free intracellular inducer concentration decreases because it binds to the repressor (cannot be measured) and because of cell division. Notably, the repressor-inducer binding cannot be measured by the HPLC-MS analytical method. Subsequently, the equilibrium between the medium and intracellular concentrations is reached upon induction at 20 μM (SS3), where partial induction still occurs. Above this concentration, full induction is achieved because all of the repressor is released from the operator sites. Therefore, for initial inducer concentration > 40 μM, [IPTG]_intra_ < [IPTG]_extra_. Our results are compatible with the assumptions concerning the IPTG concentrations in the medium and inside the biomass by Laurent and co-workers [15] and are in agreement with other theoretical hypotheses [3,14,27,28].

Figure 3 also shows a direct dependency of the measured concentration of the inducer in the medium when RhuA is at a maximum (“pseudo-steady” state) with the initial IPTG added to the reactor. However, the measured intracellular IPTG has no direct dependency on the initial inducer concentration in the “pseudo-steady” state. This fact could be due to the dynamics of the *lac* operon, as explained previously. The free intracellular inducer accumulates, and when a certain concentration is reached, intracellular IPTG can bind all of the repressor. This fact explains the intracellular depletion of the inducer. After all of the repressor is bound to the inducer, the free intracellular inducer concentration increases and accumulates when the full induction of RhuA is achieved.

**Figure 4:**
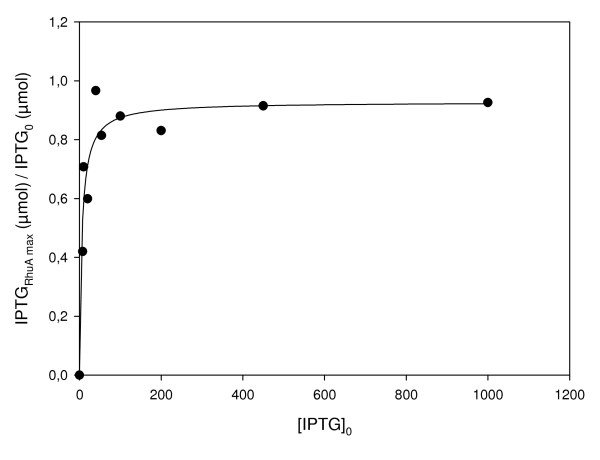
**Fraction of IPTG remaining in medium when specific RhuA activity (AU·g**^**-1**^**DCW) is maximum vs. the initial IPTG concentration (IPTG**_**0**_**, μM) added into the reactor.**

By measuring the concentration of IPTG in the medium, we also demonstrate that at any initial concentration of the inducer added to the culture, there is an excess of IPTG because the majority of it remains in the medium at “pseudo-steady” state, as shown in Figure 4. It is seen that, except upon induction at 8 μM IPTG, the majority of the inducer remains in the medium and that at high initial concentrations, only a minor amount of the inducer is taken up by the biomass.

Transitions between the non-induced and induced states were interpreted by Cohn & Horibata in their experiments with the *lac* operon. From their results, theoretical studies showed that hysteresis is an inherent property of bistable systems. However, it has been shown that *E. coli* populations were heterogeneous in terms of *lac* induction around the transition [8,16,29]. For our expression system, the bistable range (8–40 μM IPTG), where only partial induction is achieved, can be attributed to two main reasons. First, not all of the repressor is removed from the operator site in induced cells. Second, it is likely that not all of the cell population is induced due to the low availability of external IPTG. Whatever the cause, it leads to intermediate levels of RhuA activity, as shown previously in Figure 2. Additionally, from the production point of view, the maximum activity is reached with a minimal dosage of IPTG (40 μM).

**Figure 5:**
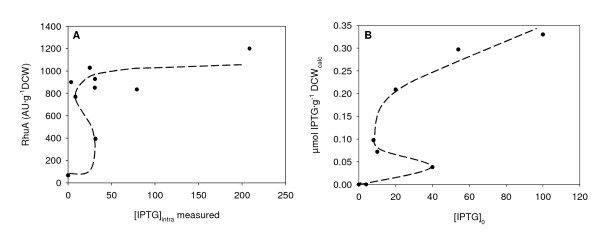
**Comparison of the “pseudo-steady” states.A)** Variation of the maximum specific RhuA activities (AU·g^-1^DCW**)** with the measured intracellular IPTG concentration (μM). **B)** Variation of the calculated intracellular inducer (μmol IPTG·g^-1^DCW) with the initial IPTG concentration (IPTG_0_, μM) added into the reactor. The analytical standard error for experimental IPTG measurements is 5 and 7% for medium and intracellular samples, respectively.

Conversely, a comparison of the “pseudo-steady” state variation has been performed. We have assessed the relationship between the amount of intracellular inducer measured with the RhuA specific activity (Figure 5A). As is seen, for similar intracellular concentrations of the inducer, different “pseudo-steady” state levels of RhuA can be attained. Furthermore, higher values of the intracellular inducer do not lead directly to higher activity values. The total inducer amount taken up by the cell has been calculated by mass balances in terms of μmol IPTG·g^-1^ DCW when RhuA is at a maximum for each initial IPTG concentration added to the reactor (Figure 5B). The behavior presented in Figure 5 could be further evidence of bistability patterns. For the non-fully induced range (IPTG ≤ 40 μM), induced and non-induced cells were expected to be present after induction. Alternatively, in this range, cell growth was not as affected by the RhuA overexpression as by the full induction. Cell growth decreases are dependent upon the initial inducer concentration for our expression system [30]. The experimental observations in this range lead to a pattern compatible with the model proposed by Noel et al. [23], which considered the effect of active transport and diffusion of the inducer. However, their simulations were based on experimental data for TMG that were obtained from Ozbudak et al. [14], in which arbitrary units for the inducer and permease activity in logarithmic scales were given. Our experimental results show great parallelisms with the simulations proposed in these studies.

### Missing inducer and estimation of inducer-repressor affinity

As indicated before, the analytical method used to quantify IPTG inside the biomass measured the free intracellular IPTG, and it was not possible to measure the inducer bound to other molecules. Therefore, we have calculated the amount of IPTG missing at each concentration level in terms of total μmol·g^-1^ DCW (data not shown). There is a lack of 0–1.2 μmol·g^-1^ DCW, which is dependent on the experiment, that represents 0-30% of the total inducer added. However, it is known that certain amounts of IPTG are bound to the repressor protein, to the permease or to other unspecific proteins. A first approach could be to assume that the inducer amount that was not measured was due to the IPTG-repressor binding. Therefore, we have estimated the inducer-repressor affinity inside the cell.

The equilibrium between free and bound inducer can be postulated as follows:

(1) $$\begin{array}{l} \mathrm{IPTG}+R⇄\mathrm{IPTG}-R\\ Kc=\frac{\left[IPTG-R\right]}{\left[IPTG\right]\cdot \left[R\right]} \end{array}$$

where

[IPTG]= = free intracellular IPTG concentration; [R]= = repressor concentration; [IPTG-R]= = inducer-repressor concentration.

and, conversely, the total repressor concentration ([R]_Total_) can be expressed as the addition of free and bound ones as follows:

(2) $$\left[R\right]+\left[IPTG-R\right]={\left[R\right]}_{\mathrm{Total}}$$

By substituting [R] from Equation 1 in to Equation 2 and assuming that [IPTG-R] can be estimated as the amount of missing intracellular inducer, as follows:

(3) $$\frac{\left[IPTG-R\right]}{\left[IPTG\right]}=\frac{IPTG_{\mathrm{intracel}}missing}{IPTG_{\mathrm{intracel}}measured}$$

**Figure 6:**
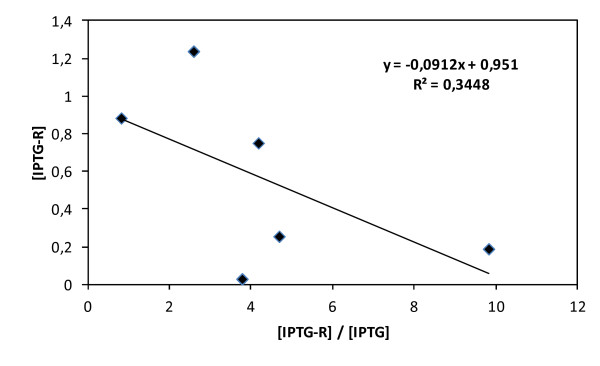
**Bound inducer concentration (calculated as inducer missing concentration) vs. the ratio between bound inducer concentration/measured inducer concentrations.** The slope corresponds to −1/k_c_. The intercept corresponds to total repressor concentration. The analytical standard error for experimental IPTG measurements is 5 and 7% for medium and intracellular samples, respectively.

By utilizing the data from all experiments, a linear relationship should be obtained that allows the determination of K_c_ and [R]_Total_. As is seen in Figure 6, the fit was not good. Nevertheless, with the necessary cautions, “rough” values of K_c_ and [*R*]_Total_ can be estimated. The inaccurate fitting could be due to the assumption that all of the missing intracellular inducer is bound to the repressor. Furthermore, it is likely that experimental error and the amount of IPTG bound to other molecules could affect the obtained result.

The dissociation constant for the repressor-inducer binding is K_c_^-1^ ≈ 4300 μM. The approximate estimation from an experimental measurement of IPTG was quite different from the ones proposed in the literature, which are 30 μM [21] or 7 – 30 μM [31]. The approximate calculated concentration of the total repressor was 430 μM, in contrast with the 0.02 μM calculated by Dunaway and co-workers. This significant difference in the order of magnitude is compatible with the higher number of LacI copies that were encoded in our expression system (pREP4 plasmid) and the higher number of repressor-operator sites encountered in the pQEαβrham plasmid in contrast with the strains studied by the other authors. From our results, we suggest a 10:1 inducer-repressor stoichiometry of induction, which is much higher than that suggested by Oehler and co-workers, indicating that our expression system is tightly regulated. However, the K_c_ and total repressor values obtained in this work were not only approximated but also overestimated because the inducer is likely bound to other molecules. It is worth noting that the order of magnitude is compatible with the calculated intracellular inducer concentration for the full induction range (see Additional file 1). Alternatively, a more rigorous treatment of this approximation would require taking into account the operator-repressor and inducer-repressor-operator bindings [17,18]. Additionally, as stated previously, our results are not comparable with those from the literature because we are dealing with a complex expression system to produce RhuA differently than wild type strains.

## Conclusions

For the first time, the induction behavior of a *lac*-derived operon was assessed using experimental data for IPTG at biomass concentrations employed to produce recombinant proteins. In the bistable range, in which a partial induction occurs, intermediate levels of RhuA activity were achieved. Full induction was achieved at IPTG concentrations greater than 40 μM. There was a direct relationship between the initial inducer concentrations and the initial inducer transport rate together with the specific activity. A majority of the inducer remains in the medium to reach equilibrium with the intracellular level. The intracellular inducer accumulation could be a further evidence of bistability of the *lac* operon and its inherent hysteresis. A comparison of our results with data from the literature was not a trivial task because there was no method available for quantifying IPTG and we utilized an expression system for recombinant protein production at high cell density cultures. Therefore, extensive studies of the inducer transport under different conditions (i.e., operation conditions or biomass concentrations) or using different strains could be of interest. To summarize, this study on IPTG transport provides experimental data for developing mathematical models that are able to predict the distribution profiles of an inducer together with the production of recombinant proteins that are of industrial interest.

## Methods

### Media, chemicals and other reagents

LB (Lennox Broth) medium, which is composed of 10 g L^-1^ peptone, 5 g L^-1^ yeast extract and 10 g L^-1^ NaCl, was used for the pre-cultures. A stock solution of ampicillin (100 mg mL^-1^; Sigma) was prepared in 50% (v/v) ethanol, filter sterilized through a Millex®GS 0.22-μm filter from Millipore and stored at -20^0^ C. Isopropyl β-D-1-thiogalactoryranoside was purchased from Sigma-Aldrich, and a stock solution was prepared by dissolving 2.38 g in 100 mL of Milli-Q water that was filter sterilized through a Millex®GS 0.22-μm filter from Millipore to obtain a stock solution of 100 mM and stored at -20^0^ C. The composition of the Defined Medium (DM) for the shake flask cultures and fed-batch fermentations were described elsewhere [32]. Formic acid (98% purity) was supplied from Panreac, and Milli-Q water was employed for the HPLC analysis.

### Strain, fermentation and growth conditions

The *E. coli* M15Δ*glyA* [pREP4] pQEαβrham expression system was used as the parental strain for RhuA production. Construction of the pQEαβrham vector for the production of RhuA under the control of the strong promoter T5 was described elsewhere [33]. This expression system is based on glycine auxotrophy to ensure plasmid stability and avoid antibiotic supplementation. A detailed explanation of the fed-batch substrate-limiting cultures can be found elsewhere [32,34]. A defined medium with glucose as the sole carbon source (20 g·L^-1^) was used for the batch phase. Once the substrate was consumed, a fed-batch phase starts supplying a feedstock solution to maintain a constant and specific growth rate of 0.22 h^-1^ by using a predefined exponential feeding profile. The overexpression of RhuA was pulse induced at 20 or 40 g DCW·L^-1^ with different initial IPTG concentrations. Table 2 shows the eleven experiments that were performed under the same conditions, with only the initial inducer concentration and biomass concentration at induction time varying.

**Table 2:** The fed-batch fermentations performed at different inducer concentration levels and the biomass concentration at induction time

**Fermentation code**	**[Inducer]**_**0**_**(μM)**
FB1^a^	8
FB2 ^a^	10
FB3 ^a^	20
FB4 ^a^	40
FB5 ^a^	54
FB6 ^a^	100
FB7 ^a^	200
FB8 ^a^	450
FB9 ^a^	1000
F3B^b^	20
F6B^b^	100

^a^ Experiments induced at 20 g DCW·L^-1^.

^b^ Experiments induced at 40 g DCW·L^-1^.

The volume of the cells was calculated according to the biomass concentration and by assuming a volume of 0.0023 L g^-1^ DCW [35] to enable the calculation of IPTG concentration inside the biomass.

### Analytical methods

The *E. coli* growth was monitored by optical density measurements at a wavelength of 600 nm using a spectrophotometer (Uvicon 941 Plus, Kontrol). Samples were diluted with distilled water until the final OD_600nm_ value was within the range 0.3-0.9. The biomass was expressed as dry cell weight (DCW), with 1 OD_600nm_ being equivalent to 0.3 g·L^-1^ DCW [36]. The cell disruption and quantitation of RhuA activity protocols are described elsewhere [37]. The IPTG analysis was performed on a Shimadzu Prominence liquid chromatograph with a UV/Vis detector operating at a wavelength of 210 nm, which was coupled to a mass spectrometer Shimadzu 2010A equipped with an ESI (electrospray ionization) interface and single quadrupole, and using a LC-10 AD solvent delivery system (pump A and B). A Shimadzu FCV-20 H2 valve unit was used to divert the flux. The injection was made with a Shimadzu SIL-10 AD automatic injector, and the data analysis was processed with Lab Solutions 3.04 software. Samples were kept in the autoinjector at room temperature. The sample preparation and chromatographic and mass spectrometer conditions are described elsewhere [24].

### Determination of IPTG concentration in fermentation samples

For medium samples, the concentration of the inducer measured by HPLC-MS was multiplied by the dilution factor. To obtain the total μmol of IPTG in the medium, the concentration was multiplied by the volume of medium inside the reactor, which can be determined by the difference between the measured total volume and the volume of the cells. The volume of the cells was calculated from the biomass concentration and by employing a specific volume of 0.0023 L·g^-1^ DCW [35]. The biomass concentration was calculated by measuring OD_600nm_ and knowing that 1 OD_600nm_ has an equivalence of 0.3 g DCW·L^-1^[36].

The intracellular samples were adjusted to an OD_600nm_ of 4 before cell disruption and subsequent IPTG analysis was performed. The intracellular concentration of the inducer could be obtained from the IPTG analysis as the biomass concentration and the equivalence to the DCW were known. The total intracellular IPTG in terms of μmol could also be determined because the biomass concentration was known and the cell volume could be assumed.

For the range of IPTG concentrations studied, the associated standard error was 5 and 7% for the medium and intracellular samples, respectively. The development and validation of IPTG analysis in medium and intracellular fed-batch fermentations was previously validated under the FDA (Food and Drug Administration) guidelines [24].

## Competing interests

The authors declare that they do not have any competing interests.

## Authors’ contributions

AFC contributed to all of the experiments and manuscript preparation. GC and JLS participated in the research design and manuscript preparation. All of the authors have read and approved the final manuscript.

## Supplementary Material

Additional file 1 Distribution profiles of IPTG in the medium and intracellular fed-batch samples together with biomass evolution and RhuA production of the nine experiments performed under different initial inducer concentration. Time zero corresponds to the induction time. Click here for file
