# Supplementary material for: Direct measurements of IPTG enable analysis of the induction behavior of E. coli in high cell density cultures
Source: Microb Cell Fact. 2012 May 9;11:58. doi: 10.1186/1475-2859-11-58 (PMC3442970; doi:10.1186/1475-2859-11-58)
Supplement: Additional file 1 — Distribution profiles of IPTG in the medium and intracellular fed-batch samples together with biomass evolution and RhuA production of the nine experiments performed under different initial inducer concentration. Time zero corresponds to the induction time. [file 1475-2859-11-58-S1.docx]

Please, find in this document the measured IPTG distribution profiles of the nine fermentations induced with IPTG when 20 g DCW·L-1 were achieved.

Figure 5.4 IPTG distribution profiles in medium in terms of amount (µmol) along with the biomass evolution (A) and in terms of concentration (µM) (B) after induction of RhuA overexpression with 8 µM IPTG. Solid line indicates the fitted curve. Fermentation code: FB1

**Figure 5.5** IPTG distribution profiles inside the biomass in terms of amount (µmol·g^-1^DCW) along with the specific RhuA production (A) and in terms of concentration (µM) (B) after induction of RhuA overexpression with 8 µM IPTG. Fermentation code: FB1

**Figure 5.6** Calculated IPTG distribution profiles inside the biomass in terms of amount (µmol·g^-1^DCW) along with the specific RhuA production (A) and in terms of concentration (µM) (B) after induction of RhuA overexpression with 8 µM IPTG. Fermentation code: FB1

**Figure 5.7** IPTG distribution profiles along time after induction of RhuA overexpression with IPTG 10 µM. (A) in medium in terms of amount (µmol) along with the biomass evolution, (B) in terms of concentration (µM) and the obtained fitted curve, (C) inside the biomass in terms of amount (µmol·g^-1^DCW) along with the specific RhuA production, (D) inside the biomass in terms of concentration (µM) and, (E) calculated inside the biomass in terms of amount (µmol·g^-1^DCW) along with the specific RhuA production and (F) calculated inside the biomass in terms of concentration (µM). Fermentation code: FB2

**Figure 5.8** IPTG distribution profiles along time after induction of RhuA overexpression with IPTG 20 µM. (A) in medium in terms of amount (µmol) along with the biomass evolution, (B) in terms of concentration (µM) and the obtained fitted curve, (C) inside the biomass in terms of amount (µmol·g^-1^DCW) along with the specific RhuA production, (D) inside the biomass in terms of concentration (µM), (E) calculated inside the biomass in terms of amount (µmol·g^-1^DCW) along with the specific RhuA production and (F) calculated inside the biomass in terms of concentration (µM). Fermentation code: FB3

**Figure 5.9** IPTG distribution profiles along time after induction of RhuA overexpression with IPTG 40 µM. (A) in medium in terms of amount (µmol) along with the biomass evolution, (B) in terms of concentration (µM) and the obtained fitted curve, (C) inside the biomass in terms of amount (µmol·g^-1^DCW) along with the specific RhuA production, (D) inside the biomass in terms of concentration (µM), (E) calculated inside the biomass in terms of amount (µmol·g^-1^DCW) along with the specific RhuA production and (F) calculated inside the biomass in terms of concentration (µM). Fermentation code: FB4

**Figure 5.10** IPTG distribution profiles along time after induction of RhuA overexpression with IPTG 54 µM. (A) in medium in terms of amount (µmol) along with the biomass evolution, (B) in terms of concentration (µM) and the obtained fitted curve, (C) calculated inside the biomass in terms of amount (µmol·g^-1^DCW) along with the specific RhuA production and (D) calculated inside the biomass in terms of concentration (µM). Fermentation code: FB5. Note: *Intracellular samples could not be analyzed and some of the medium samples were missed.*

**Figure 5.11** IPTG distribution profiles along time after induction of RhuA overexpression with IPTG 100 µM. (A) in medium in terms of amount (µmol) along with the biomass evolution, (B) in terms of concentration (µM) and the obtained fitted curve, (C) inside the biomass in terms of amount (µmol·g^-1^DCW) along with the specific RhuA production, (D) inside the biomass in terms of concentration (µM), (E) calculated inside the biomass in terms of amount (µmol·g^-1^DCW) along with the specific RhuA production and (F) calculated inside the biomass in terms of concentration (µM). Fermentation code: FB6

**Figure 5.12** IPTG distribution profiles along time after induction of RhuA overexpression with IPTG 200 µM. (A) in medium in terms of amount (µmol) along with the biomass evolution, (B) in terms of concentration (µM) and the obtained fitted curve, (C) inside the biomass in terms of amount (µmol·g^-1^DCW) along with the specific RhuA production, (D) inside the biomass in terms of concentration (µM), (E) calculated inside the biomass in terms of amount (µmol·g^-1^DCW) along with the specific RhuA production and (F) calculated inside the biomass in terms of concentration (µM). Fermentation code: FB7

**Figure 5.13** IPTG distribution profiles along time after induction of RhuA overexpression with IPTG 450 µM. (A) in medium in terms of amount (µmol) along with the biomass evolution, (B) in terms of concentration (µM) and the obtained fitted curve, (C) inside the biomass in terms of amount (µmol·g^-1^DCW) along with the specific RhuA production, (D) inside the biomass in terms of concentration (µM), (E) calculated inside the biomass in terms of amount (µmol·g^-1^DCW) along with the specific RhuA production and (F) calculated inside the biomass in terms of concentration (µM). Fermentation code: FB8

**Figure 5.14** IPTG distribution profiles along time after induction of RhuA overexpression with IPTG 1000 µM. (A) in medium in terms of amount (µmol) along with the biomass evolution, (B) in terms of concentration (µM) and the obtained fitted curve, (C) inside the biomass in terms of amount (µmol·g^-1^DCW) along with the specific RhuA production, (D) inside the biomass in terms of concentration (µM), (E) calculated inside the biomass in terms of amount (µmol·g^-1^DCW) along with the specific RhuA production and (F) calculated inside the biomass in terms of concentration (µM). Fermentation code: FB9
